# Sirtuins mediate mitochondrial quality control mechanisms: a novel therapeutic target for osteoporosis

**DOI:** 10.3389/fendo.2023.1281213

**Published:** 2024-01-08

**Authors:** Tianchi Zhang, Lining Wang, Xiping Duan, Yuanyuan Niu, Muzhe Li, Li Yun, Haitao Sun, Yong Ma, Yang Guo

**Affiliations:** ^1^ Laboratory of New Techniques of Restoration & Reconstruction, Institute of Traumatology & Orthopedics, Nanjing University of Chinese Medicine, Nanjing, Jiangsu, China; ^2^ School of Chinese Medicine, School of Integrated Chinese and Western Medicine, Nanjing University of Chinese Medicine, Nanjing, Jiangsu, China; ^3^ Acupuncture Anesthesia Clinical Research Institute, Yueyang Hospital of Integrated Traditional Chinese and Western Medicine, Shanghai University of Traditional Chinese Medicine, Shanghai, China; ^4^ Department of Orthopedic, Wuxi Huishan District People’s Hospital, Wuxi, Jiangsu, China; ^5^ Department of Traumatology and Orthopedics, Affiliated Hospital of Nanjing University of Chinese Medicine, Nanjing, Jiangsu, China

**Keywords:** osteoporosis, sirtuins, mitochondrial quality control, mitochondrial dysfunction, bone

## Abstract

Mitochondria plays a role in cell differentiation and apoptosis processes. Maintaining mitochondrial function is critical, and this involves various aspects of mitochondrial quality control such as protein homeostasis, biogenesis, dynamics, and mitophagy. Osteoporosis, a metabolic bone disorder, primarily arises from two factors: the dysregulation between lipogenic and osteogenic differentiation of aging bone marrow mesenchymal stem cells, and the imbalance between osteoblast-mediated bone formation and osteoclast-mediated bone resorption. Mitochondrial quality control has the potential to mitigate or even reverse the effects. Among the Sirtuin family, consisting of seven Sirtuins (SIRT1-7), SIRT1-SIRT6 play a crucial role in maintaining mitochondrial quality control. Additionally, SIRT1, SIRT3, SIRT6, and SIRT7 are directly involved in normal bone development and homeostasis by modulating bone cells. However, the precise mechanism by which these Sirtuins exert their effects remains unclear. This article reviews the impact of various aspects of mitochondrial quality control on osteoporosis, focusing on how SIRT1, SIRT3, and SIRT6 can improve osteoporosis by regulating mitochondrial protein homeostasis, biogenesis, and mitophagy. Furthermore, we provide an overview of the current state of clinical and preclinical drugs that can activate Sirtuins to improve osteoporosis. Specific Sirtuin-activating compounds are effective, but further studies are needed. The findings of this study may offer valuable insights for future research on osteoporosis and the development of clinical prevention and therapeutic target strategies.

## Graphical Abstract

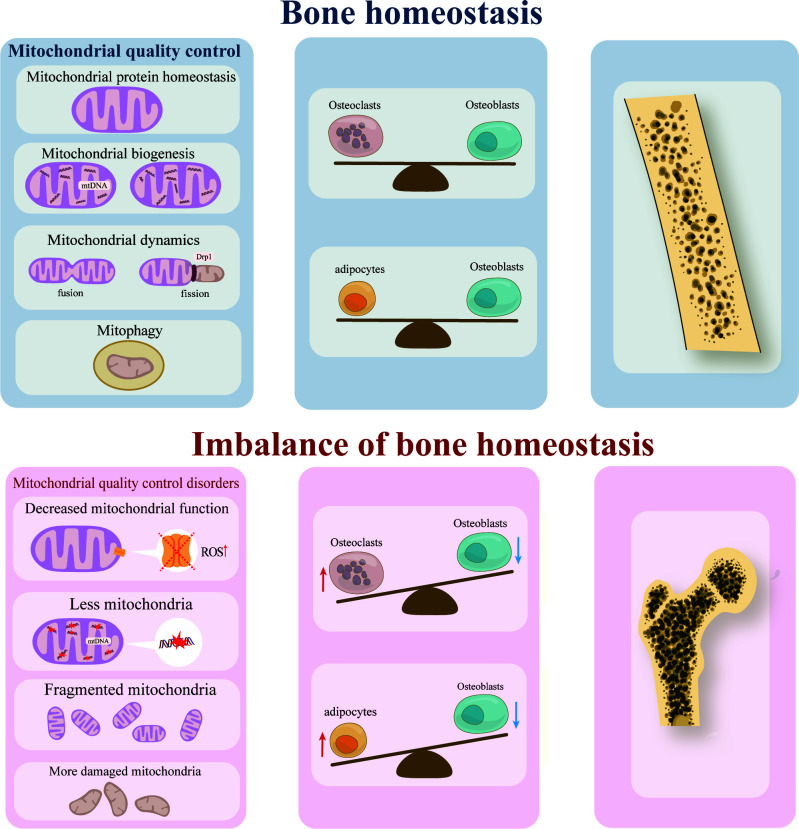

## 1 Introduction

Osteoporosis (OP) is a systemic skeletal disorder characterized by reduced bone mass, compromised bone microarchitecture, heightened bone fragility, and susceptibility to fractures. At the cellular level, OP primarily arises from two factors: the dysregulation between lipogenic and osteogenic differentiation of aging bone marrow-derived mesenchymal stem cells (BMSCs), and the imbalance between osteoblast-mediated bone formation and osteoclast-mediated bone resorption. Cellular senescence in the bone microenvironment plays an important role in the occurrence and development of OP. Delaying the aging process of terminal cells such as osteoblasts and osteocytes involved in bone metabolism can slow disease progression (1, 2), but this effect is not sufficient to improve overall bone homeostasis. The number of BMSCs in bone tissue of OP patients is reduced and senescent, while the self-renewal ability of senescent BMSCs is weakened, the number is decreased, and the osteogenic differentiation is weakened, and the adipogenic differentiation is enhanced, leading to the failure of bone tissue to recruit enough osteoblasts to meet the needs of osteogenesis (3). Currently, the primary therapeutic target for OP is nuclear factor-κb(NF-κb, RANKL), which promotes bone resorption. The representative drugs for osteoporosis treatment encompass bisphosphonates, which effectively inhibit the activity of NF-κb; however, they are associated with gastrointestinal reactions and potential nephrotic syndrome (4). Denosumab, a novel therapeutic drug, has demonstrated superior efficacy in alleviating pain and enhancing patients’ quality of life (5). However, its potential implementation may be impeded by its substantial cost, posing financial challenges for both patients and healthcare providers. Furthermore, the therapeutic targets for osteoblasts and bone marrow mesenchymal stem cells are currently focused on Wnt, with drugs in stages of clinical and preclinical trials. Consequently, further investigation into the mechanism of OP and the exploration of more effective therapeutic targets are needed.

Mitochondria are integral to maintaining the equilibrium of bone resorption and formation. The bone marrow environment is characterized by low oxygen levels. During the differentiation of BMSCs, the mode of energy metabolism needs to switch from anaerobic glycolysis to mitochondrial energy metabolism (6). Consequently, the quantity, functionality, structure, and metabolic byproducts of mitochondria significantly impact the proliferation and differentiation of BMSCs, osteoblasts, and osteoclasts during the metabolic adaptation process. In the context of bone formation, the diminished quantity and anomalous structure of mitochondria can result in reduced osteogenic potential and an elevated level of lipid differentiation in BMSCs. Conversely, the disruption of mitochondrial structure and the heightened production of reactive oxygen species (ROS) can enhance osteoclast activity, thereby facilitating bone resorption (7–9). The regulation of mitochondrial quality, including mitochondrial protein homeostasis, biogenesis, and mitophagy, plays a crucial role in maintaining mitochondrial function and overall bone health.

The Sirtuin family, consisting of seven members (SIRT1-7), plays a crucial role in regulating various aspects of mitochondrial quality control. Specifically, SIRT1-SIRT6 have been shown to modulate mitochondrial quality control mechanisms (10). Additionally, sirtuins have been found to have numerous beneficial effects, including the regulation of caloric restriction, which can lead to a decreased risk of cancer, cardiovascular diseases, nervous system diseases, and bone metabolism diseases (11–14). Notably, the expression of SIRT1, SIRT3, SIRT6, and SIRT7 has been implicated in the regulation of bone homeostasis and may serve as a potential target for the treatment of OP (15). However, the specific sirtuin and aspect of mitochondrial quality control that contribute to this enhanced effect have yet to be definitively determined. Consequently, this article undertakes a comprehensive examination of the impacts of diverse facets of mitochondrial quality control on osteoporosis, as well as the influence of SIRT1, SIRT3, and SIRT6 on osteoporosis through the regulation of mitochondrial quality control. Furthermore, we provide a summary of the present state of clinical and preclinical medications that activate Sirtuins to ameliorate osteoporosis. The findings of this study may offer valuable insights for future research on osteoporosis and the development of clinical prevention and therapeutic target strategies.

## 2 Mitochondrial quality control and osteoporosis

Mitochondria in their normal state possess the ability to uphold cellular homeostasis and exert influence on neighboring cells. In cases where mitochondria within recipient cells exhibit signs of aging or damage, the introduction of mitochondria or mitochondrial DNA from healthy BMSCs can reinstate cellular viability (6). Consequently, the preservation of an optimal mitochondrial quality serves as the fundamental prerequisite for sustaining cellular homeostasis, tissue stability, and overall bodily function equilibrium. In summary, the regulation of mitochondrial quality holds potential in the prevention and treatment of OP.

### 2.1 Mitochondrial protein homeostasis

**Figure 1:**
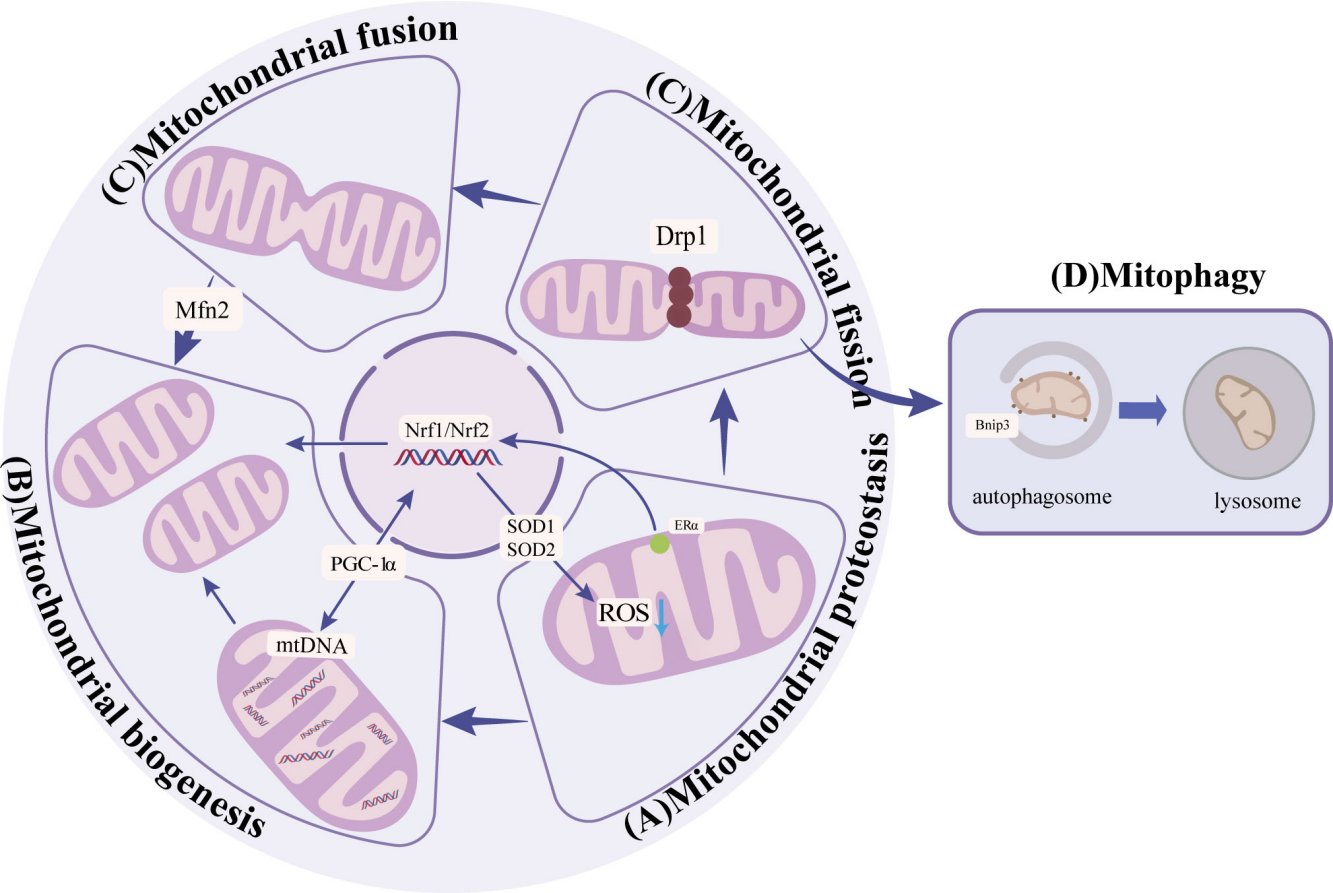
**(A)** Mitochondrial protein homeostasis: Activation of SIRT3-FOXO3a-SOD2 pathway to remove ROS and ERα-NRF1-HTRA2/proteasome pathway to remove damaged proteins to maintain mitochondrial protein homeostasis. **(B)** Mitochondrial biogenesis:PGC-1α promotes the generation of new mitochondria by activating NRF1, NRF2, and other nuclear factors. **(C)** Mitochondrial dynamics: Mfn2 promotes mitochondrial outer membrane fusion, and Drp1 promotes mitochondrial fission. **(D)** Mitophagy: through receptor-mediated BNIP3 ubiquitin-mediated and PINK1/Parkin pathways.

Mitochondrial protein homeostasis is essential for maintaining mitochondrial function and decreasing the level of reactive oxygen species (ROS) (16). Homeostasis requires regulating a series of enzymes to lower ROS levels and remove unfolded or damaged proteins. Mitochondrial protein homeostasis activates mitochondrial unfolded protein response(UPRmt)and other pathways (17) to induce transcription of nuclear and mitochondrial genes to enhance protein folding capacity, thereby preventing harmful protein accumulation in mitochondria (16, 17)(**Figure 1**). Among them, promoting the SIRT3-Forkhead boxO3(FOXO3a)-Superoxide dismutase2(SOD2) pathway can directly accelerate the decomposition of ROS and reduce protein damage (18). In addition, activation of transcription of estrogen receptor α(Erα)- nuclear respiratory factors (NRFs)-HTRA2(HtrA serine peptidase 2)/protease clears unfolded protein or excess protein (17). When antioxidant enzymes cannot clear ROS in time, high levels of ROS will lead to the death of osteoblasts and osteocytes, thus inhibiting the bone formation and resulting in reduced bone structure (19). On the other hand, high ROS levels can also enhance the activity of osteoclasts and promote bone resorption, further worsening bone condition (7–9). Mitochondrial dysfunction and structural damage can cause ROS leakage and further damage mitochondrial structure, causing a vicious cycle (**Table 1**). Therefore, the activation of signaling pathways, such as the mitochondrial unfolded protein response, is crucial for maintaining homeostasis of mitochondrial proteins and thereby enhancing the prevention of osteoporosis.

**Table 1:** Aspects of mitochondrial quality control and related molecular pathways in osteoporosis.

Aspects	Molecular Pathways	Effects on osteoporosis
Mitochondrial protein homeostasis	SOD2↓	Osteoblasts and osteocytes↓ Osteoclast activity↑ (7–9) (19)
	ERα-NRF1-HTRA2/proteasome↓	
Mitochondrial biogenesis	PGC-1α↑	Osteogenic differentiation↑ (3)
Mitochondrial dynamics	Mfn2↑ Drp1↑	Osteogenic potential↑ (20)
		Osteoblast-differentiated BMSCs↓ (20)
	Mitofilin↓	Osteogenic potential↓ (21)`````````
Mitophagy	BNIP3↓PINK1/Parkin↓	BMSC senescence↑ Osteogenic differentiation↓ Adipogenic differentiation↑ (22)

↓ means decrease, ↑ means increase

### 2.2 Mitochondrial biogenesis

Mitochondrial biogenesis increases the number and quality of mitochondria to adapt to energy metabolism (23). Mitochondrial biogenesis includes nuclear and mitochondrial genome transcription, synthesis of proteins and lipids, and assembly of the electron transfer chain. Damage to any step may affect mitochondrial energy metabolism (24). The primary regulator in this process is peroxisome proliIerators-activated receptor γ coactivator alpha α).PGC-1α responds to the increased energy demand by activating nuclear factors such as NRF1、NRF2, promoting new production of mitochondrial, or regulating mitochondrial DNA replication and transcription to promote mitochondrial protein synthesis (25–27) (**Figure 1**). The expression of PGC-1a is significantly diminished in osteocytes and cells surrounding bone trabeculae in aged mice (3). Further studies showed that in PGC-1a knockout mice, the accumulation of adipocytes in the bone marrow cavity may be the cause (3). The accumulation of fat inhibits the osteogenic differentiation of BMSCs and promotes the adipogenic differentiation (**Table 1**). These results suggest that PGC-1α influences mitochondrial biogenesis, which is indispensable in bone homeostasis. n the experiment involving MC3T3-E1 cells, the supplementation of drugs to enhance PGC-1α has been found to enhance mitochondrial ATP production, increase mitochondrial membrane potential, and decrease ROS levels, thereby leading to an improvement in osteogenic differentiation (28). Furthermore, PGC-1β has also been identified as a contributor to mitochondrial biogenesis in addition to PGC-1α. In the context of osteoclast proliferation and differentiation, the upregulation of PGC-1β expression promotes mitochondrial biogenesis (29). This finding suggests that PGC-1β may have a detrimental impact on bone homeostasis. Immunofluorescence analysis revealed a significant reduction in PGC-1β expression within the bone tissue of OVX mice (30). *In vitro* experiments demonstrated that lentivirus-mediated silencing of the PGC-1β gene in RAW264.7 cells resulted in decreased numbers of mature osteoclasts (31). These results indicate the important influence of mitochondrial biogenesis on bone homeostasis.

### 2.3 Mitochondrial dynamics

Mitochondrial dynamics encompass various processes such as fusion, division, mobility, and cristae remodeling, which collectively alter the structure of mitochondria to adapt to fluctuations in energy metabolism (32, 33). In undifferentiated bone marrow mesenchymal stem cells, mitochondria are located near the nucleus and appear as fragmented spheres (34, 35). However, upon differentiation into osteoblasts, mitochondria disperse evenly throughout the cytoplasm, adopting an elongated branching network configuration (34, 35). Subsequent investigations have revealed that the differentiation of bone marrow mesenchymal stem cells into osteoblasts is influenced by factors related to mitochondrial fusion and fission. During the initial phase of osteogenesis, the interconnected tubular mitochondrial network undergoes development, resulting in an enhanced energy change. This process is accompanied by an up-regulation of the mitochondrial fusion protein (Mfn2), which plays a crucial role in the fusion of mitochondrial outer membranes (20, 36); Conversely, the absence of Mfn2 impedes the fusion of mitochondria, leading to a reduction in oxygen consumption, energy changes, and ultimately, the loss of osteogenic potential (20). These findings suggest that the expression of Mfn2 may serve as a promoter for the osteogenic differentiation of BMSCs (37)(**Figure 1**). In contrast, the osteogenic BMSCs exhibited a higher degree of mitochondrial fusion and a decreased expression of mitochondrial fission factors, such as dynamin-related protein (Drp1) and Fission1 (Fis1), when compared to the chondrogenic BMSCs (20) (**Figure 1**). Previous research has demonstrated that the suppression of Drp1 expression can effectively restore impaired osteogenic differentiation in the presence of oxidative stress, indicating that the upregulation of Drp1 and other related factors to inhibit mitochondrial fission may serve as a potential intervention strategy for osteoporosis (38). Taken together, these findings suggest that the promotion of mitochondrial fusion and the inhibition of mitochondrial fission have a positive influence on osteogenic differentiation (**Table 1**). However, no studies have observed the changes in mitochondrial morphology and the expression of related factors during osteoclast differentiation, which needs to be supplemented.

In terms of ultrastructure, mitochondria can enhance energy metabolism through the alteration of cristae shape and density, thereby regulating the assembly and stability of the electron transport chain (39). Evidence has shown that ridge morphology plays a significant role in osteogenic differentiation (21). Mitofilin plays an essential role in cristae morphology. When mitofilin was absent, the mitochondrial membrane potential of BMSCs from SAMP6 mice was impaired, resulting in the formation of swollen and inefficient mitochondria, which leads to the reduction of osteogenic markers (21). However, supplementation of mitofilin reversed the above molecular effects and slowed down the senescence of SAMP6 mice BMSCs. This study suggests that it is feasible to intervene in OP by regulating the expression of mitofilin to improve the morphology of the cristae (**Table 1**). However, more effective studies are needed. Therefore, mitochondrial dynamics can play a crucial role in BMSCs differentiation by affecting the mitochondrial structure and cristae morphology. Regulating these factors may have a positive significance in improving osteogenic differentiation in OP.

### 2.4 Mitophagy

Mitophagy is typically executed via the ubiquitin-mediated PINK1/Parkin pathway and the receptor-mediated BNIP3 pathway. Dysfunctional and excessive mitochondria are selectively engulfed by autophagosomes and subsequently degraded in lysosomes (40) (**Figure 1**). PINK1, a serine kinase, accumulates in the outer mitochondrial membrane upon depolarization of the inner membrane. This accumulation facilitates the recruitment of Parkin to the mitochondrial surface, leading to ubiquitination of mitochondrial proteins, aggregation of p62, and specific binding of LC3, thereby facilitating the process of mitophagy (41). BNIP3 typically exists as an inactive monomer within the cytoplasm, but in response to stress signals, it undergoes a transformation into stable homodimers and integrates into the OMM (42). This integration of BNIP3 into the OMM facilitates the accumulation of PINK1 on the OMM, thereby promoting mitophagy (43). Following mitochondrial injury, the accumulation of ROS and the disruption of the electrical potential across the membrane result in a loss of the potential gradient between the inner and outer mitochondrial membranes, ultimately leading to a decrease in mitophagy. The process of mitophagy, in turn, exerts an influence on bone metabolism by impacting osteoblasts, osteoclasts, and BMSCs (44).

The involvement of PINK1/Parkin-mediated mitophagy in OP is a crucial determinant. A study revealed a decrease in the expression of PINK1 among OP patients (45). Additionally, in mouse models with PINK1 gene deletion following ovariectomy, a significant reduction in bone mass was observed (45). Furthermore, osteoblasts exhibiting low levels of PINK1 expression exhibited a significant decrease in the expression of osteogenic markers, such as ALP and OCN (45). Consequently, it can be inferred that mitochondrial autophagy mediated by PINK1 plays a pivotal role in promoting osteoblast differentiation and mitigating bone mass loss. In experiments with advanced glycation end products (AGEs) intervention, BMSCs senescence was significantly accelerated (22), the mechanism may be that AGEs inhibit mitophagy and aggravate oxidative stress in a concentration-dependent manner. *In vitro* studies, autophagy marker LC3B and mitophagy associated protein Parkin increased with the increase of AGEs concentration, while autophagy substrate P62 increased. Targeted improvement of mitophagy can significantly alleviate the aging of BMSCs (22), which confirmed the importance of mitophagy for bone marrow mesenchymal stem cell homeostasis.

On the contrary, the induction of mitophagy can also facilitate the differentiation of BMSCs into osteoblasts while inhibiting their differentiation into adipocytes (22). The process of mitophagy, mediated by BNIP3, has a significant impact on osteoporosis. In osteoclasts obtained from mice lacking SIRT3, the acetylation of PINK1 increased while the levels of Bnip3 decreased (46). Glucocorticoid (GC; specifically dexamethasone, DEX)-induced osteonecrosis of the femoral head poses a complex challenge in the field of orthopedics. Overexpression of HIF-1α demonstrated resistance against DEX-induced apoptosis in a hypoxic environment. The down-regulation of HIF-1α and BNIP3 was observed upon exposure to DEX, indicating that the protective influence of HIF-1α on bone is mediated through BNIP3 (47). In ovariectomized mice, the researchers observed a decrease in the levels of BNIP3, while the overexpression of BNIP3 in osteoclast precursor cells led to a reduction in osteoclast production (48). Consequently, the promotion of mitophagy can potentially retard the aging of bone marrow mesenchymal stem cells and facilitate osteogenic differentiation, thereby decelerating the progression of osteoporosis (**Table 1**).

## 3 Sirtuins improve osteoporosis through the mitochondrial pathway

Sirtuins, which are proteins dependent on NAD+, are distributed in the nucleus, cytoplasm, and mitochondria, and they are involved in the deacetylation modification or ribosylation of adenosine diphosphate (ADP) (49). Sirtuins are implicated in the regulation of cellular senescence. Previous studies have reported that Sirtuins possess the ability to delay cellular senescence in various cell types, including human endothelial cells and oxidant-exposed macrophages (50, 51). Furthermore, depletion of SIRT1 and SIRT6 has been shown to promote endothelial cell senescence (52). Conversely, overexpression of SIRT1 and SIRT6 inhibits cellular senescence in human coronary endothelial cells, primary porcine aortic endothelial cells, and lung cells (53, 54) Additionally, SIRT1 regulates stem cell senescence and is crucial for maintaining stem cell self-renewal (55, 56). While expression levels of SIRT1 are high in embryonic stem cells but gradually decrease in differentiated cells (57), it has been demonstrated that overexpression of SIRT1 delays BMSCs senescence (58). Moreover, the mitochondrial sirtuin known as SIRT3 exhibits high expression levels in HSCs suggesting its potential role in preventing stem cell senescence as well (59). By modulating aging-related gene expression associated with bone metabolism through regulation of the NF-κb signaling pathway (60), it has been demonstrated that SIRT6 plays a regulatory role. Notably, decreased expression levels were observed for both members of the sirtuin family; specifically reduced expression was found for femoral neck tissue from elderly patients regarding their level of expression for Sirt1 while decreased expression was observed for both bone tissue and BMSCs from elderly patients regarding their level of expression for SIRT6. These studies provide compelling evidence supporting an indispensable role played by the sirtuin family during age-related mechanisms leading to bone loss.

Previous research has demonstrated that SIRT1, SIRT2, SIRT3, SIRT6, and SIRT7 have a significant impact on bone development and homeostasis by directly influencing bone cells (5) (61). Specifically, Sirt1 plays a crucial role in chondrocytes and osteocytes for normal bone development and homeostasis, while Sirt3, Sirt6, and Sirt7 also contribute to bone homeostasis (62). The significance of SIRT1-SIRT6 in maintaining mitochondrial quality control has been well-established (16). Consequently, this study aims to comprehensively examine and assess the roles of SIRT1, SIRT3, and SIRT6 in both bone metabolism regulation and mitochondrial quality control.

### 3.1 SIRT1

**Figure 2:**
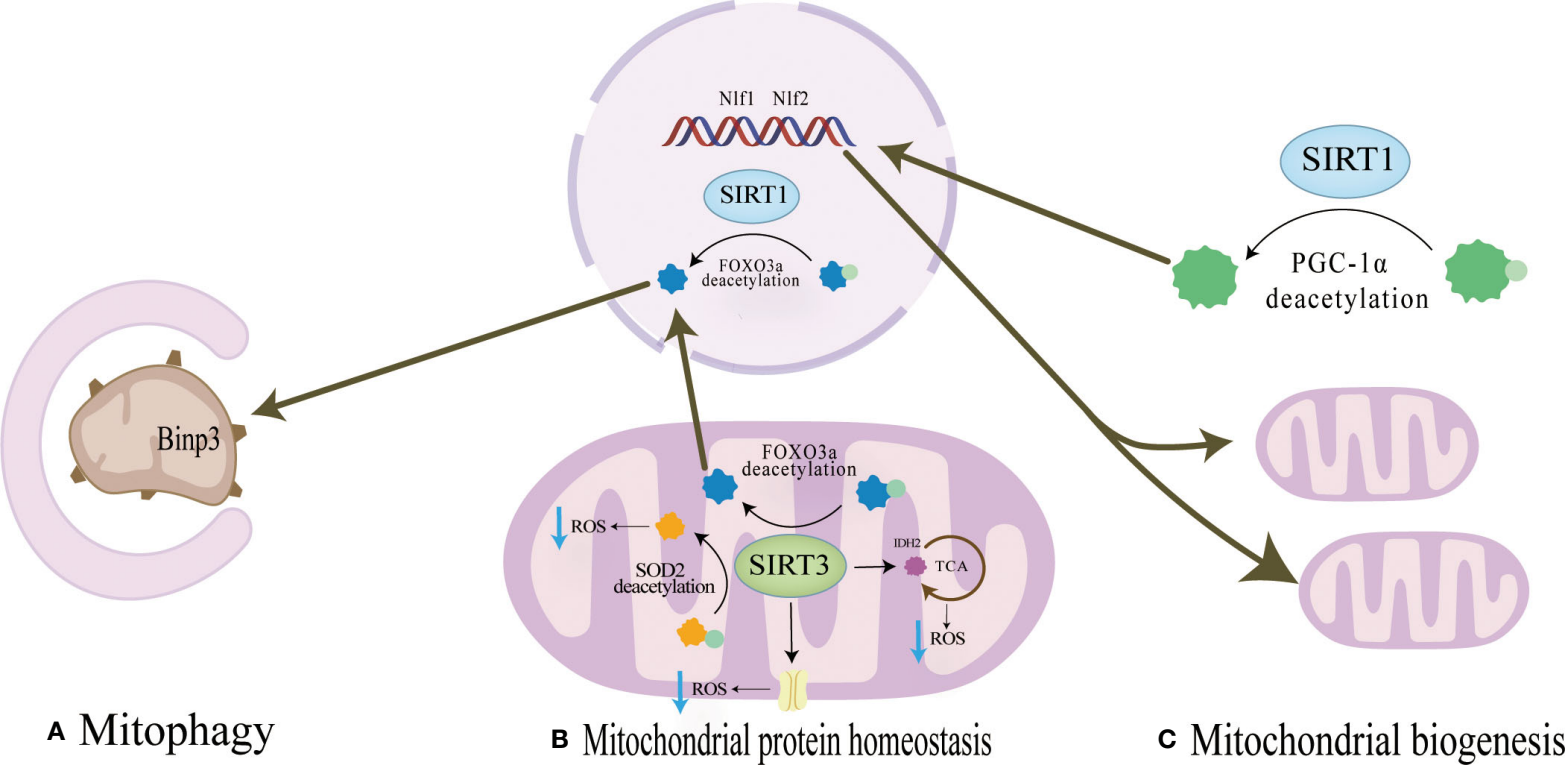
**(A)** Mitochondrial protein homeostasis:SIRT3 can directly induce ROS scavenging by deacetylating SOD2,and the activation of FOXO3a by SIRT1 or SIRT3 promotes the production of SOD2 or catalase in the nucleus and mitochondria and reduce ROS levels. **(B)** Mitophagy SIRT1 promotes mitophagy by deacetylating FOXO3a to promote BNIP3 expressionSIRT3 promotes expression of BNIP3 by deacetylating FOXO3a, while SIRT3 ameliorates mitophagy. **(C)** Mitochondrial biogenesis SIRT1 can directly deacetylate PGC-1α and make PGC-1α more easily enter the nucleus, which directly promotes mitochondrial biogenesis and increases the number and activity of mitochondria.

SIRT1 is mainly located in the nucleus and cytoplasm and regulates the expression of transcription factors such as p53, nuclear factor-κb (NF-κB), and FOXOs deacetylation modification of histone and non-histone proteins such as H3, H,4 and H1 (63).SIRT1 regulates mitochondrial protein homeostasis by promoting antioxidant enzymes to reduce ROS levels, thereby regulating bone homeostasis in bone resorption and formation(**Figure 2**). Activation of FOXO3a by SIRT1 can promote the production of SOD2 or catalase in the nucleus and mitochondria and reduce ROS levels (64–66). These responses inhibited the proliferation of osteoclast precursor cells and slowed down the generation of osteoclasts in mice (67, 68). In terms of bone formation, SIRT1 expression could promote osteogenic differentiation of BMSCs (69) and SIRT1 could also inhibit the adipogenic differentiation of BMSCs (70); in addition, it can reduce the apoptosis of osteoblasts (71); these effects were also carried out by increasing the production of antioxidant enzymes such as SOD1 and SOD2 (**Table 2**). Interestingly, there were gender differences in the phenotype of SIRT1 regulation of bone homeostasis. The possible reason is that in the process of scavenging mitochondrial ROS levels, SIRT1 increases PGC-1α by deacetylating FOXO3a, which may activate the ERα-NRF1-HTRA2/proteasome pathway in UPRmt and crosstalk with estrogen receptor α in the mitochondrial membrane space (79), However, further studies are needed to clarify the mechanism. In addition, SIRT1 can also mediate PGC-1α to promote mitochondrial biogenesis and affect osteogenic differentiation (**Figure 2**). SIRT1 can directly deacetylate PGC-1α and make PGC-1α more easily enter the nucleus, which directly promotes mitochondrial biogenesis and increases the number and activity of mitochondria (26, 27). When osteogenic ability was inhibited, expression levels of PGC-1α and SIRT1 decreased in osteoblasts. When SIRT1 and PGC-1α were activated, mitochondrial biogenesis was increased, and the osteogenesis ability was improved (72, 80). These studies suggest SIRT1 and PGC-1α may positively affect osteogenic differentiation by promoting mitochondrial biogenesis (**Table 2**). In the absence of SIRT1, the expression level of PGC-1α decreased, and the improvement effect of PGC-1α on osteoblasts failed (72). SIRT1 is essential for mitophagy under mitochondrial stress (**Figure 2**). SIRT1 promotes mitophagy by deacetylating FOXO3a to promote BNIP3 expression (81). Without SIRT1, mitophagy could not be activated usually (81). Studies have shown that SIRT1 enhances mitophagy to improve osteoblasts and increases bone mineral density in osteoporotic rats to improve OP (73) (**Table 2**). Therefore, SIRT1 can interfere with OP by promoting mitochondrial biogenesis, reducing oxidative damage, and regulating mitochondrial quality control pathways such as mitophagy to slow down BMSCs aging, promote osteogenic differentiation, and inhibit osteoclast proliferation.

**Table 2:** Effects of sirtuins on mitochondrial quality control in osteoporosis.

Sirtuins	Mitochondrial quality control	Molecular Pathways	Effect of intervention on osteoporosis
SIRT1	Regulating mitochondrial protein homeostasis	FOXO3a↑SOD2↑	Osteogenic differentiation↑Adipogenic differentiation↓ The apoptosis of osteoblasts (64, 69–71)
			Osteoclast precursor cells↓ osteoclasts↓
	Promoting of mitochondrial biogenesis	PGC-1α↑	Osteogenic differentiation ↑ (72)
	Promoting mitophagy	FOXO3a↑BNIP3↑	Osteogenic differentiation (73)
SIRT3	Regulating mitochondrial protein homeostasis	SOD2 ↑	Primary osteoblasts differentiation ability↑ Osteoclasts generation↓ (74, 75)
		IDH2↑	
		ETC function↑	
	Promoting mitophagy	FOXO3a↑BNIP3↑	BMSCs senescence↓ Osteogenic potential ↑ Adipogenic differentiation↓ (22, 76)
SIRT6	Regulating mitochondrial protein homeostasis	SIRT3↑	Bone resorption↓ (77)
	Promoting mitophagy		BMSCs senescence↓ Osteogenic differentiation↑ (78)

↓ means decrease, ↑ means increase

### 3.2 SIRT3

SIRT3 can reduce ROS and maintain mitochondrial protein homeostasis by activating mitochondrial unfolded protein responses (**Figure 2**). SIRT3 is located in mitochondria and has unique advantages in regulating ROS. Firstly, SIRT3 can directly induce ROS scavenging by deacetylating SOD2 (82, 83). In addition, SIRT3 functions by improving ETC (84–89) or affecting isocitrate dehydrogenase (IDH2), a-promoting ketoglutarate production in the TCA cycle to indirectly reduce ROS levels (90, 91). In addition to the direct effects on ROS levels, in response to the UPRmt, SIRT3 can control ROS levels by activating FOXO3a, which is encoded by FOXO3a, to regulate nuclear and mitochondrial production of SOD2 and catalase (65, 66). The effects of SIRT3 on bone homeostasis are dual-sided. Huh et al. demonstrated that mice lacking SIRT3 at 8 weeks of age exhibited osteoporosis, indicating an active role of Sirt3 in peak bone mass development (92). Additionally, SIRT3 is crucial for osteoclast mitochondrial activity and bone resorption during the progression of osteoporosis. Osteoclasts deficient in SIRT3 display impaired capacity for bone resorption (93) Mice overexpressing SIRT3 show increased adipocytes in the tibia, while BMSCs with SIRT3 overexpression exhibit enhanced adipogenic differentiation potential. Moreover, overexpression of SIRT3 promotes osteoclast formation, resulting in a 2.5-fold increase in the number of osteoclasts on the bone surface (94) Further investigation is warranted to explore the resistance to bone loss after estrogen deficiency due to OVX observed in SIRT3−/− mice (95).

The SIRT3/SOD2 axis plays a crucial role in maintaining the balance of reactive oxygen species (ROS) levels, which in turn affects osteoblast differentiation, bone formation, and osteoclast generation (74). The SIRT3/SOD2 axis plays a crucial role in maintaining the balance of reactive oxygen species (ROS) levels, which in turn affects osteoblast differentiation, bone formation, and osteoclast generation (74). Additionally, SIRT3 knockout mice exhibit significant osteopenia accompanied by dysfunctional osteoblasts (74). Conversely, overexpression of SOD2 or SIRT3 can enhance the differentiation ability of primary osteoblasts from SIRT3-deficient mice, while inhibiting SIRT3 expression can lead to increased osteoclast activity (74, 75) (**Table 2**). he impact of SIRT3 expression on bone mass exhibits gender disparities. Similar to SIRT1, the SIRT3-FOXO3a axis has the potential to augment peroxisome proliferator-activated PGC-1α in reaction to oxidative stress, thereby potentially activating the ERα-NRF1-HTRA2/proteasome pathway in UPRmt. Additionally, it may engage in reciprocal interactions with estrogen receptor α within the mitochondrial membrane compartment (79).

SIRT3 plays a crucial role in promoting mitochondrial mitophagy, as depicted in **Figure 2**, thereby decelerating the aging process of BMSCs and facilitating their osteogenic differentiation(**Figure 2**). SIRT3 promotes expression of BNIP3 by deacetylating FOXO3a (76), while SIRT3 ameliorates mitophagy, which can alleviate the senescence of BMSCs to improve OP. Mitophagy is decreased in senescent BMSCs, and promoting mitophagy can reduce the expression of senescence-related proteins such as p16, p21, and p53 in BMSCs. The accumulation of AGEs will further cause mitochondrial dysfunction, inhibit mitochondrial phagocytosis with the increase of age concentration, and aggravate the aging of BMSCs (22). SIRT3 knockout reduced mitophagy and accelerated BMSCs senescence. In osteoclasts from SIRT3-deficient mice, the acetylation of PINK1 increased and the levels of Bnip3 decreased (46). This suggests that the effect of SIRT3 on osteoclast differentiation and formation may be carried out by affecting mitophagy. Revised sentence: Overexpression of SIRT3 enhances mitophagy, reduces the expression of senescence-related proteins p16, p21, and p53, effectively mitigates BMSCs senescence in SAMP6 mice, augments the osteogenic potential while diminishing adipogenic potential of BMSCs, and improves OP (22) (**Table 2**). *In vitro* knockdown of SIRT3 inhibits mitochondrial biogenesis and osteoclast differentiation; however, knockdown of SIRT3 increases trabecular bone mass in female mice due to impaired osteoclast production. Although osteoclast progenitors from aged Sirt3-null mice can differentiate into osteoclasts, these differentiated cells exhibit reduced oxidative phosphorylation and mitophagy as well as impaired absorptive activity (46). Nevertheless, treatment with the Sirt3 inhibitor LC-0296 restores the effects on osteoclast formation and mitochondrial function while increasing bone mass in aging mice (94). Therefore, it is evident that SIRT3 exerts dual effects on OP by alleviating oxidative damage and regulating mitophagy.

### 3.3 SIRT6

SIRT6 predominantly localizes within the nucleus and plays a crucial role in governing the response and restoration of impaired DNA. SIRT6 regulates the RelA subunit of NF-κb bymodifying the cellular senescence-related gene expression (44). SIRT6KO mice showed significant weight loss, small body size and poor bone development. SIRT6KO can also directly promote osteoclast differentiation, possibly resulting in an excess of small osteoclasts, which in turn triggers hyperactive bone resorption (96). It has been observed that SIRT6 contributes to the preservation of mitochondrial protein homeostasis and enhances osteoblasts by diminishing levels of reactive oxygen species (ROS), although the precise mechanism remains unidentified. Notably, the upregulation of SIRT6 has been demonstrated to stimulate the expression of NRF2 and SIRT3, suggesting that SIRT6’s impact on ROS regulation may be partially mediated indirectly through SIRT3 (97).The specific mechanism needs to be verified by both knockdown experiments. In osteoblasts that were overexpressed with SIRT6, it was observed that the level of ROS decreased, the inflammatory response of osteoblasts was inhibited, and bone resorption was weakened (77). Furthermore, SIRT6 was found to enhance the ability of BMSCs to regulate OP through the regulation of mitophagy. Inhibition of SIRT6 resulted in a decrease in autophagy levels and osteogenesis ability of BMSCs (78). However, the use of the autophagy activator rapamycin mitigated these adverse reactions caused by SIRT6 inhibition in BMSCs (78) (**Table 2**). In mechanism, SIRT6 regulates autophagy of BMSCs and aging and osteogenic differentiation of BMSCs through activation of AKT-mTOR pathway (78). Therefore, SIRT6 can intervene in OP by reducing ROS levels and regulating mitophagy to improve BMSCs senescence and osteogenic differentiation.

## 4 Potential drugs targeting sirtuins for the treatment of osteoporosis

Considering the impaired mitochondrial homeostasis in OP, therapeutic approaches that modulate sirtuins to regulate mitochondrial quality control could alleviate mitochondrial dysfunction and intervene in OP. The researchers were surprised to find that the mechanism of action of some clinical drugs may be through the activation of sirtuins to regulate mitochondrial quality control, and in order to achieve more stable efficacy and fewer side effects, the researchers developed more STACs. We analyzed the research progress of the effects of clinical and preclinical drugs on osteoporosis.

### 4.1 SIRT1/SIRT3 activators

#### 4.1.1 Non-specific SIRT1/SIRT3 activators

Melatonin, a drug commonly utilized in clinical settings for regulating circadian rhythm and circulation, has demonstrated positive effects in the treatment of osteoporosis (98). In cellular studies, the stimulation of SIRT1 and SIRT3 by melatonin resulted in significant increases in SOD2 and glutathione peroxidase 1(GPX1), thereby enhancing the osteogenic differentiation ability of BMSCs in postmenopausal individuals with OP (99, 100). Although melatonin did not significantly improve biochemical markers of bone formation and bone resorption in perimenopausal women, it demonstrated a concentration-dependent increase in BMD at the lumbar spine and femoral neck (101–104). However, other studies have reported doubts about the efficacy of melatonin in treating OP (105), and concerns about its side effects have been raised (106, 107).

In the realm of STACs, resveratrol was the initial generation of SIRT1 activators that was discovered in 2003 (108). Resveratrol has the ability to augment the expression of SIRT1 protein in a manner that is dependent on the dosage (109). Through experiments conducted on OP rats, it was observed that resveratrol safeguards osteoblasts by modulating the SIRT1 and PI3K/AKT/mTOR signaling pathways, thereby enhancing mitochondrial phagocytosis (73). Furthermore, resveratrol also possesses the capability to activate SIRT3 (110). The bone-preserving effects of resveratrol may be partially attributed to the involvement of SIRT3, as the absence of SIRT3 diminishes the bone-preserving effect of resveratrol (110).Clinically, resveratrol treatment for 16 weeks significantly increased bone mass in obese elderly men, according to a randomized placebo-controlled trial (111) (**Table 3**). A recent randomized controlled trial has also shown that regular resveratrol supplementation improves bone mineral density in postmenopausal women (112).

**Table 3:** Potential drugs targeting sirtuins for the treatment of osteoporosis.

Targeted Sirtuins		Drugs	Ways and effects of improving osteoporosis
SIRT1/SIRT3	non-specific SIRT1/SIRT3 activators	Melatonin	Osteogenesis of BMSCs↑ (99, 100)
		Resveratrol	Osteoblasts↑ (73)
			Bone mass in elderly obese men↑ (111)
			Bone mineral density in postmenopausal women↑ (112)
	SIRT1	SRT1720、SRT2104 and SRT3025	Osteoclast progenitor cell proliferation↓Osteoclast development↓ (64) Bone mineral density↑ (113)
	SIRT3	Zoledronic acid	Osteogenesis of BMSCs↑ (114)
		Honophenol	No experimental studies in osteoporosis
SIRT6		Metformin	Adipogenic differentiation↓ Osteogenic differentiation↑ (115–117)
		Anthocyanins	Bone formation and bone resorption↓Bone loss↓ (115)

↓ means decrease, ↑ means increase

#### 4.1.2 SIRT1-specific activators

Over the course of the past twenty years, several STACs have been identified as capable of activating SIRT1. Among these, SRT1720, SRT2104, and SRT3025 have received the most attention in research (**Table 3**). Activation of SIRT1, specifically through the use of SRT2104 and SRT3025, has been found to impede the proliferation of osteoclast progenitor cells and diminish osteoclast development in mice (64). Furthermore, the inhibition of osteoclast generation by SRT3025 was found to be ineffective in the absence of SIRT1 (64). Thus highlighting the specificity of the compound.It is worth noting that while the third-generation agonist SRT3025 demonstrated significant improvement in the ovariectomized model from cellular to *in vivo* studies, its impact on the vertebrae was particularly significant (118). However, whether the anti-osteoporosis effect of SRT3025 is induced by changes in activation of NF-B or MAPK is debatable (64). In contrast, long-term administration of SRT2104 in mice has been shown to extend lifespan and enhance bone density, while also demonstrating efficacy in preventing bone loss in more severe disused osteoporosis models (113). Based on the current performance of these drugs, we expect more preclinical and clinical trials.

#### 4.1.3 SIRT3-specific activators

From a clinical perspective, the administration of zoledronic acid serves to inhibit bone resorption, thereby mitigating the progression of osteoporosis. This effect is believed to be achieved through the inhibition of oxidative stress via the SIRT3/SOD2 pathway, as well as the promotion of osteogenesis in BMSCs (114) (**Table 3**). On the other hand, honokiol, a naturally occurring bisphenol compound derived from magnolia bark, possesses various beneficial properties such as anti-inflammatory, antioxidant, anti-tumor, and neuroprotective effects. Notably, honokiol has the ability to bind to SIRT3 and enhance its expression and activity (119, 120). In the context of heart failure, honokiol has been shown to delay disease progression by activating SIRT3, thereby safeguarding against mitochondrial damage and cellular death. But no experimental studies have explored the role of honokiol in OP model (**Table 3**).

### 4.2 SIRT6-specific activators

Anthocyanins have demonstrated efficacy as activators of SIRT6, resulting in increased expression and activity (121, 122). A recent study conducted on ovariectomized menopausal mice revealed that anthocyanin treatment effectively prevented bone loss by inhibiting osteoclast formation and bone resorption (115) (**Table 3**). Further investigations on anthocyanins in alternative osteoporosis models are warranted to assess their therapeutic potential in preventing bone loss, potentially leading to future clinical trials.

In a clinical observation, it was determined that metformin has the potential to decrease the risk of OP regardless of other variables, such as diabetes (116). Specifically, metformin has the ability to mitigate oxidative stress in BMSCs, thereby impeding lipogenic differentiation and promoting osteogenic differentiation (117). Meanwhile, Zheng et al (123) and Wang et al (124) discovered that the absence of SIRT6 significantly diminishes the beneficial effects of metformin on osteogenesis. These findings indicate that SIRT6 plays a pivotal role in the anti-osteoporotic properties of metformin, necessitating further investigation.

Regarding STACs, anthocyanins have demonstrated effectiveness as activators of SIRT6, leading to enhanced expression and activity (121, 122). Notably, a recent animal investigation exhibited that anthocyanin treatment effectively hindered osteoclast formation and bone resorption, thereby preventing bone loss in ovariectomized menopausal mice (115) (**Table 3**). However, further exploration of anthocyanins in alternative osteoporosis models is imperative to assess their therapeutic potential in mitigating bone loss, potentially culminating in future clinical trials.

## 5 Research trends

**Figure 3:**
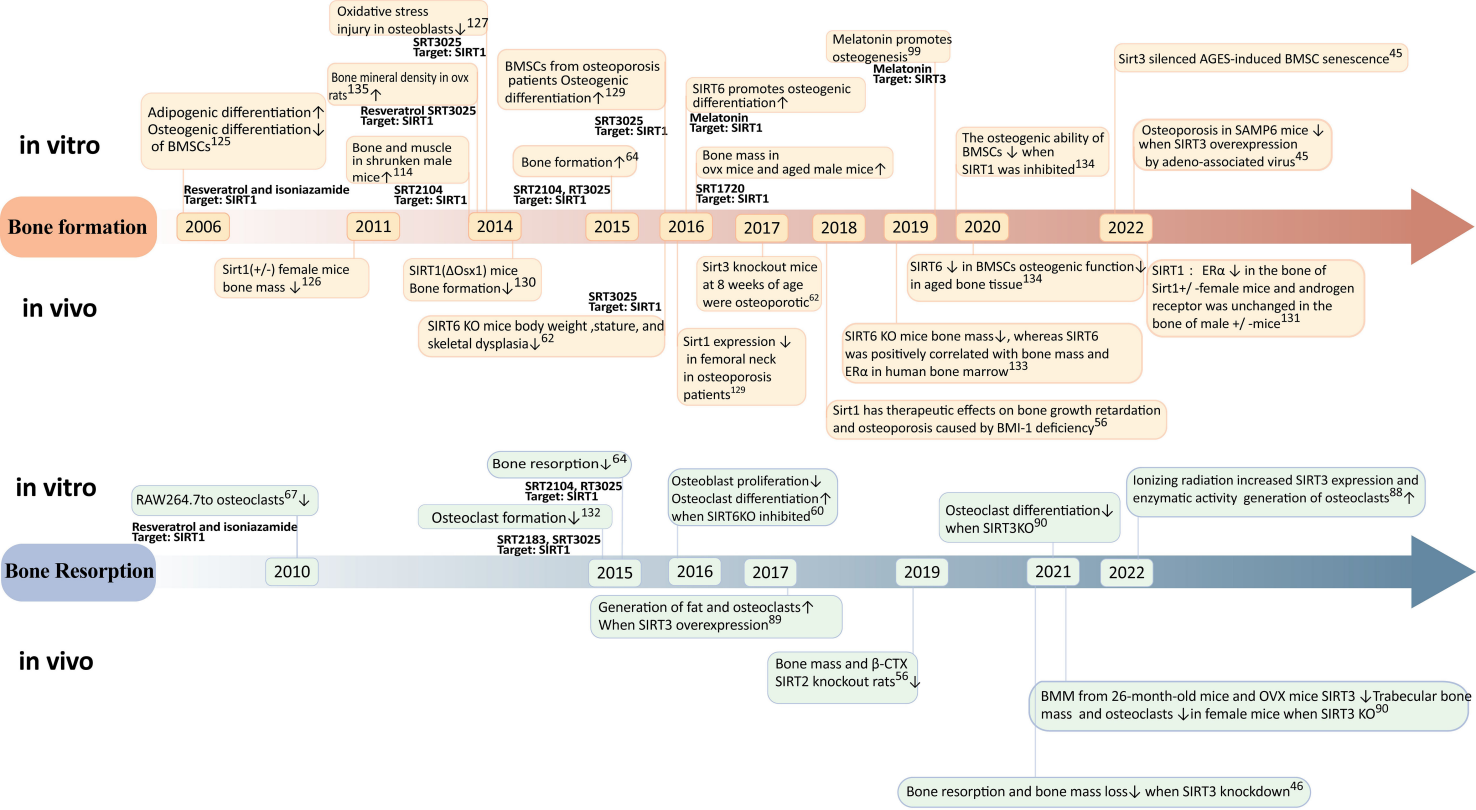
Timeline of progress in sirtuins research. Reratrol and T3025 rescue decreased bone mineral density in ovx rats (134). SIRT3reduces mitochondrial oxidative stress and mtDNA damage in osteoblasts (135). Melatonin promotes osteogenic differentiation (136).

**Figure 4:**
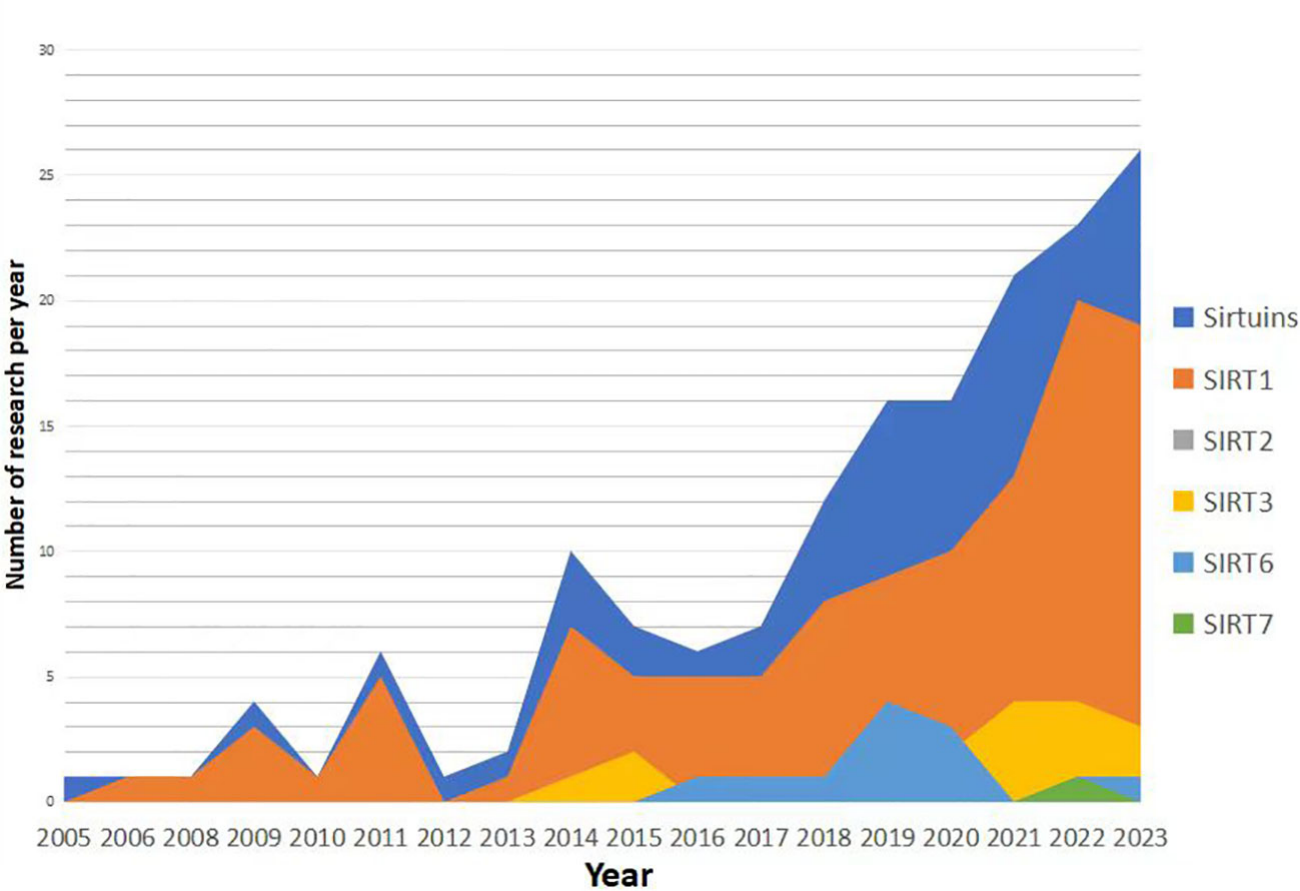
Number of research per year.

Research trends in the relationship between Sirtuins and osteoporosis have shown a gradual increase since 2005 (**Figures 3**, **4**), with a primary focus on the impact of Sirtuins on osteogenesis, while fewer studies have explored their effect on osteoclasts. Among these investigations, there is a predominant emphasis on SIRT1 and its association with osteoporosis, followed by attention toward SIRT3 and SIRT6. Further investigation is required to elucidate the connection between SIRT7 and osteoporosis.

The impact of SIRT1 on osteoporosis has been extensively investigated. In 2006, cellular experiments demonstrated that the activation of SIRT1 under the influence of resveratrol could suppress lipogenic differentiation in BMSCs and enhance osteogenic differentiation (125). Female mice with Sirt1(+/-) exhibited significantly reduced bone mass in 2011, providing evidence for the crucial role of sirtuin-1 in bone formation (126). In 2014, the specific activator of SIRT1, known as SRT3025, was utilized to rescue bone mass in postmenopausal osteoporosis model rats and cost-type atrophic osteoporosis model mice (113, 118, 127). Furthermore, a study conducted in 2016 confirmed the expression of SIRT1 in the femoral neck region of patients with osteoporosis (128), thereby validating specific activation of SIRT1 as a potential therapeutic target for treating this condition. Further investigations into the mechanism involving targeted knockout of bone cell-specific sirtuins have revealed gender differences regarding the treatment effects on osteoporosis mediated by SIRT1 (129). Specifically, estrogen receptor alpha (ERα) levels were decreased in bones from female mice with heterozygous deletion (+/-)of Sirt11 gene while androgen receptor(AR) remained unchangedin male mice with heterozygous deletion (+/-) (130). It is suggested that ERα may regulate cortical bone response to sirtuin-activated pathways (130). Additionally,in terms of bone resorption,SIRT1 activation can inhibitosteoclastogenesis (67). A dual effect was observed when using an activator for treating osteoporosis; it reducesbone resorption while promotingbone formation (131).

Sirt3 may exert a dual role in osteoporosis through diverse pathways. Regarding bone formation, SIRT3 has been demonstrated to mitigate mitochondrial oxidative stress and mtDNA damage *in vitro* in osteoblasts In 2017, sirt3 knockout mice exhibited the onset of osteoporosis at 8 weeks of age (92), while the overexpression of sirt3 via intravenous injection of recombinant adeno-associated viruses carrying sirt3 plasmids significantly ameliorated osteoporosis in SAMP6 mice in 2020. These findings suggest that Sirt3 might enhance osteogenic differentiation during early stages of life (22). However, concerning bone resorption, the upregulation of SIRT3 in 2017 promoted adipogenesis and osteoclastogenesis (94). Moreover, elevated levels of SIRT3 were observed in primary bone marrow-derived macrophages from 26-month-old mice and OVX mice in 2021 (95). Knockdown experiments revealed that impaired osteoclast production due to reduced SIRT3 expression led to increased trabecular bone mass specifically in female mice. Additionally, the inhibition or knockout of SIRT3 mitigated augmented bone resorption and loss induced by estrogen deficiency both *in vivo* using LC-0296 as a specific inhibitor for SIRT3 and through genetic manipulation (46) Collectively, these results indicate that during aging, SIRT3 may promote lipid differentiation, osteoclast differentiation, and bone resorption within the context of mesenchymal stem cells derived from the bone marrow ultimately culminating into skeletal deterioration. Overall, it is crucial to further investigate the age- and sex-dependent dual effects exerted by Sirt3 on osteoporosis.

SIRT6 has a positive effect on bone metabolism. 2016 SIRT6 knockout mice exhibit significant weight loss, short stature, and skeletal dysplasia; meanwhile, SIRT6KO inhibits osteoblast proliferation and differentiation and induces osteoclast differentiation (60). Further study found that sirtuin 6 knockout mice had reduced bone mass in 2019 *in vivo*, and sirtuin 6 was positively correlated with human bone marrow bone mass and ERα (132). In 2020, the expression of SIRT6 in BMSC is decreased, and the osteogenic function in aging bone tissue is decreased. *In vitro*, the osteogenic capacity of BMSCs decreased after SIRT6 inhibition (133).

## 6 Conclusions and discussion

This article reviews the role of sirtuins in improving osteoporosis through the regulation of mitochondrial quality control. Specifically, we examines the targets and signaling pathways associated with mitochondrial quality control in both *in vitro* and *in vivo* models of osteoporosis. The study highlights the significance of mitochondrial protein homeostasis, mitochondrial biogenesis, mitochondrial dynamics, and mitochondrial autophagy in the progression of osteoporosis, as these processes directly affect BMSCs, osteoblasts, and osteoclasts. Research in the field of mitochondrial quality control primarily focuses on various targets such as SOD2, Erα, PGC-1a/β, Mfn2, Drp1, Fis1, Mitofilin, BNIP3, PINK1, Parkin. The regulation of these targets by SIRT1, SIRT3, and SIRT6 can influence the aging, differentiation, and apoptosis of BMSCS, osteoblasts, and osteoclasts, consequently impacting mitochondrial quality control. Additionally, numerous drugs have been identified that can activate sirtuin, thereby enhancing mitochondrial quality control and potentially improving OP.

The process of maintaining mitochondrial quality involves multiple interconnected components, with particular emphasis on mitochondrial dynamics and mitophagy. Disruption of the molecules involved in mitochondrial dynamics hinders the proper functioning of mitophagy. Recent investigations have revealed that the division of mitochondria into distinct sizes serves as a foundation for selective autophagy. Additionally, current research on the mitochondrial pathway to enhance osteoporosis primarily concentrates on its impact on osteogenic differentiation lineage. The process of osteoclast differentiation lineage, which involves the sequential differentiation of hematopoietic stem cells into osteoclasts, is accompanied by alterations in cellular metabolism due to the high energy demands of osteoclast resorption. Currently, the primary factor influencing osteoclast differentiation is ROS on osteoclast differentiation (137). Additional research is required to investigate the impact of enhancing mitochondrial quality on the osteoclast lineage, as well as the co-culture of osteogenic and osteoclast lineages. Finally, it is noteworthy to mention that there exists only a single published article that investigates the potential of enhancing mitochondrial dynamics for the regulation of osteoporosis. This scarcity may be attributed to the fact that the assessment of mitochondrial morphology is not commonly employed as a benchmark in experimental observations. Nevertheless, it has been extensively studied in the context of cardiac and neurological disorders. The modulation of mitochondrial morphology actively contributes to the transformation of BMSCs. Therefore, the importance of investigating the impact of enhancing mitochondrial dynamics on the regulation of osteoporosis necessitates further experimentation and observation.

Sirtuins can impact various aspects of mitochondrial quality control. For instance, SIRT1 directly deacetylates PGC-1α, while SIRT3 regulates the expression of FOXO3a to activate PGC-1α. Furthermore, sirtuins exhibit interplay among themselves, with SIRT3 and SIRT6 mutually enhancing each other’s expression. Consequently, future investigations should not only focus on the individual expression of sirtuin genes about bone homeostasis but also consider the potential effects of simultaneously inhibiting the expression of multiple sirtuins. It is worth noting that SIRT1, SIRT3, and SIRT6 exert distinct influences on sexual characteristics. The potential mechanisms underlying the differential impacts of SIRT1 and SIRT3 on bone loss associated with sex-related factors may involve the modulation of reactive oxygen species (ROS) levels and the mitigation of ROS-induced oxidative stress, thereby regulating osteoporosis. The up-regulation of foxo3a triggers an interplay between PGC-1α and the ERα-NRF1-HTRA2/proteasome pathway in UPRmt. Mice overexpressing SIRT6 exhibit resistance to postmenopausal bone loss induced by ovariectomy. In contrast to SIRT1 and SIRT3, SIRT6 acts as a downstream factor of foxo3a, and its impact on sex-related bone loss may be mediated through the activation of the SIRT6-AMPK-PGC-1α pathway, which interacts reciprocally with the ERα-NRF1-HTRA2/proteasome pathway in UPRmt. Furthermore, the study provided further evidence to support the crucial role of PGC-1α in the regulation of mitochondrial mass by sirtuins. Notably, apart from cells associated with bone homeostasis, alterations in the expression of sirtuins in muscle and the neuronal system were found to impact bone mass. Specifically, a decline in SIRT1 expression in the muscle soleus of aged transgenic mice resulted in elevated levels of ROS, ultimately leading to a reduction in bone mass (138). Additionally, neuronal SIRT1 was found to decrease bone mass by enhancing sympathetic nervous system signaling (139).

The potential role of drug activators, such as zoledronic acid, in enhancing mitochondrial oxidative stress via the SIRT3/SOD2 signaling pathway to promote osteogenesis is a subject of interest in current clinical drug mechanisms. Preclinical investigations have demonstrated that targeted activation of sirtuins can effectively modulate the equilibrium between osteoblasts and osteoclasts within the bone microenvironment, leading to a significant improvement in bone mineral density in experimental animals. While the clinical and experimental findings regarding sirtuins activators are promising, further research is needed to determine their practical application in a clinical setting.

## Author contributions

TZ: Writing – original draft. LW: Writing – review & editing. XD: Visualization, Writing – original draft. YN: Investigation, Writing – original draft. ML: Writing – review & editing. LY: Writing – review & editing. HS: Writing – review & editing. YG: Writing – review & editing. YM: Writing – review & editing.
